# Ressuscitação Cardiopulmonar Extracorpórea na América Latina: O Desafio Vai Além do Financiamento

**DOI:** 10.36660/abc.20260240

**Published:** 2026-04-24

**Authors:** Luiz Fernando Caneo

**Affiliations:** 1 Instituto do Coração do Hospital das Clínicas da Faculdade de Medicina da Universidade de São Paulo São Paulo SP Brasil Instituto do Coração do Hospital das Clínicas da Faculdade de Medicina da Universidade de São Paulo, São Paulo, SP – Brasil

**Keywords:** Reanimação Cardiopulmonar, Oxigenação por Membrana Extracorpórea, Parada Cardíaca

Quando a ressuscitação cardiopulmonar (RCP) convencional falha em restaurar a circulação após uma parada cardíaca, a janela de sobrevivência com um desfecho neurológico favorável se reduz rapidamente. A ressuscitação cardiopulmonar extracorpórea (ECPR) surgiu como uma estratégia potencial para prolongar essa janela, estabelecendo rapidamente o suporte cardiopulmonar por meio de oxigenação por membrana extracorpórea venoarterial (ECMO-VA). De acordo com a *Extracorporeal Life Support Organization* (ELSO), a ECPR é definida como o início rápido da ECMO-VA em pacientes com parada cardíaca refratária que não obtêm retorno sustentado da circulação espontânea por meio da ressuscitação convencional.^1^

A ECPR tornou-se uma estratégia de resgate consolidada para parada cardiorrespiratória refratária, tanto em ambientes hospitalares (PCR-IH) quanto em casos selecionados de ambientes extra-hospitalar (PCR-EH) encaminhados a centros especializados. Os desfechos após PCR diferem substancialmente entre esses contextos. Pacientes com PCR-IH geralmente apresentam melhor sobrevida e melhores resultados neurológicos do que aqueles com PCR-EH, principalmente devido ao reconhecimento precoce, RCP imediata de alta qualidade e acesso rápido a intervenções avançadas, como desfibrilação e monitoramento invasivo. Por outro lado, a PCR-EH está frequentemente associada a atrasos no reconhecimento e tratamento, intervalos prolongados de ausência ou baixo fluxo sanguíneo e variabilidade no atendimento pré-hospitalar. A intervenção oportuna e o acesso rápido a terapias avançadas continuam sendo determinantes críticos para a sobrevida e a recuperação neurológica.

Apesar dessas vantagens, a PCR-IH continua sendo um desafio clínico significativo. Somente nos Estados Unidos, mais de 290.000 adultos sofrem PCR-IH anualmente,² com mortalidade persistentemente alta e risco substancial de lesão neurológica entre os sobreviventes. A ECPR visa restaurar a perfusão sistêmica quando a RCP convencional falha, preservando a função dos órgãos enquanto a causa subjacente é identificada e tratada. Estudos observacionais e registros indicam que, para certos pacientes, como aqueles com parada cardíaca presenciada, causas reversíveis e curtos períodos de baixo fluxo, a ECPR pode melhorar a sobrevida em comparação com a ressuscitação convencional.^3,4^

O uso da ECPR cresceu mundialmente, mas as evidências que a sustentam ainda são limitadas e variadas. A maioria dos dados provém de centros especializados na América do Norte, Europa e Ásia Oriental. A implementação de um programa de ECPR exige mais do que simplesmente dispor de equipamentos e circuitos de ECMO. As recomendações destacam a necessidade de um forte comprometimento institucional, coordenação multidisciplinar, envolvimento administrativo, avaliação de viabilidade e critérios claros de seleção de pacientes.^1^ Esses programas demandam equipe treinada, infraestrutura dedicada e experiência contínua para garantir uma resposta rápida. Programas de ECPR bem-sucedidos geralmente se baseiam em centros de ECMO consolidados, com equipes experientes, protocolos bem definidos e treinamento contínuo. A ECPR é a camada mais complexa do suporte extracorpóreo avançado — a cereja do bolo de um programa de ECMO já estabelecido. Atender a esses requisitos é um desafio para muitos sistemas de saúde da América Latina, onde poucos centros possuem a estrutura, a experiência e os recursos necessários.

Nesse contexto, Burgos et al.^5^ fornecem informações valiosas sobre a implementação da ECPR em um cenário latino-americano. Em sua coorte retrospectiva unicêntrica, os autores relatam os desfechos de pacientes adultos submetidos a ECMO-VA para PCR-IH ao longo de nove anos em um centro cardiovascular de alta complexidade na Argentina. Embora a coorte seja relativamente pequena, incluindo quinze pacientes, os resultados relatados são notáveis. Os autores observaram que 60% dos pacientes puderam ser retirados da ECMO, 46% sobreviveram à internação, e os sobreviventes apresentaram boa recuperação neurológica, sem mortes adicionais durante o acompanhamento.

Esses resultados estão em consonância com os relatados em registros internacionais, onde a sobrevida após ECPR normalmente varia entre 30% e 40%.^6^ Notavelmente, esses resultados foram alcançados em um ambiente de saúde com menos recursos do que os de países de alta renda, reforçando a ideia de que o sucesso do programa depende não apenas da tecnologia, mas também da organização e da eficiência do sistema. Um ponto forte do estudo é a descrição detalhada da infraestrutura que dá suporte ao programa de ECPR. Os autores descrevem uma equipe multidisciplinar de ECMO, disponível 24 horas por dia, que inclui cirurgiões cardiovasculares, cardiologistas, intensivistas, perfusionistas, enfermeiros e outros profissionais. Essa estrutura organizacional permitiu a implementação de protocolos padronizados para seleção de pacientes, canulação, manejo da anticoagulação e cuidados pós-ressuscitação. A organização baseada em sistemas é cada vez mais vista como um fator principal nos resultados da ressuscitação extracorpórea. O tempo até o estabelecimento do fluxo em ECMO, ou a duração do baixo fluxo, é um importante preditor de sobrevida e recuperação neurológica.^7^ Esse tempo só pode ser reduzido por meio de uma logística institucional bem estruturada e amplamente treinada.

Os resultados relatados por Burgos et al.^5^ devem ser interpretados com cautela. O desenho retrospectivo, a ausência de um grupo controle e o pequeno tamanho da amostra limitam conclusões definitivas sobre a eficácia comparativa da ECPR. Além disso, a coorte foi altamente selecionada, incluindo apenas pacientes com parada cardíaca presenciada, ritmo inicial chocável e etiologia cardíaca provável — características associadas, de forma independente, a melhores desfechos, independentemente da estratégia de ressuscitação. As taxas relativamente altas de complicações, incluindo sangramento, infecção e lesão renal aguda, ressaltam a complexidade do manejo da ECPR. Embora essas complicações sejam esperadas e estejam de acordo com relatos anteriores, elas enfatizam a importância da seleção criteriosa de pacientes e do cuidado multidisciplinar meticuloso para garantir que os benefícios da ECPR superem os riscos.

Além dos resultados clínicos, este estudo oferece informações importantes para o desenvolvimento de programas de ECPR na América Latina e em outras regiões com recursos limitados. O suporte circulatório avançado é frequentemente percebido como inacessível em países de baixa e média renda devido aos custos e às necessidades de infraestrutura. No entanto, a experiência apresentada por Burgos et al.^5^ sugere que programas bem estruturados em centros de referência podem superar algumas dessas barreiras. A concentração de conhecimento especializado, o treinamento da equipe e a implementação de protocolos padronizados podem permitir que centros selecionados ofereçam terapias avançadas com resultados adequados.

Em resumo, Burgos et al.^5^ demonstram a viabilidade da ECPR em um contexto latino-americano. Apesar das limitações inerentes a um pequeno estudo observacional, suas descobertas sugerem que resultados comparáveis aos padrões internacionais podem ser alcançados em centros especializados com programas estruturados de ECMO, mesmo em ambientes com recursos limitados. À medida que a ressuscitação extracorpórea evolui, o progresso dependerá não apenas dos avanços tecnológicos, mas também do desenvolvimento de sistemas de atendimento coordenados, da seleção rigorosa de pacientes e do compromisso institucional contínuo.

Em última análise, a ECPR deve ser vista não apenas como uma intervenção tecnológica, mas também como uma terapia sistêmica que integra diagnóstico rápido, ressuscitação especializada e atendimento multidisciplinar.

A experiência apresentada neste estudo reforça uma lição fundamental para programas emergentes de ECPR: apesar da relevância da tecnologia e dos recursos financeiros, o sucesso depende, sobretudo, de liderança, organização, treinamento e comprometimento institucional. Em última análise, o desafio não se resume à falta de recursos financeiros, mas à construção de sistemas capazes de entregar excelência quando cada minuto é determinante.
