# Supplementary material for: Ressuscitação Cardiopulmonar Extracorpórea na América Latina: O Desafio Vai Além do Financiamento
Source: Arq Bras Cardiol. 2026 Apr 24;123(3):e20260240. [Article in Portuguese] doi: 10.36660/abc.20260240 (PMC13128212; doi:10.36660/abc.20260240)
Supplement: Translation

# Translation: Extracorporeal Cardiopulmonary Resuscitation in Latin America: It is Not All about the Money

**DOI:** 10.36660/abc.20260240i

**Authors:** Luiz Fernando Caneo

**Affiliations:** 1 São Paulo SP Brazil Instituto do Coração do Hospital das Clínicas da Faculdade de Medicina da Universidade de São Paulo, São Paulo, SP – Brazil

**Keywords:** Cardiopulmonary Resuscitation, Extracorporeal Membrane Oxygenation, Heart Arrest

When conventional cardiopulmonary resuscitation fails to restore circulation after cardiac arrest, the window for survival with a favorable neurological outcome narrows rapidly. Extracorporeal cardiopulmonary resuscitation (ECPR) has emerged as a potential strategy to prolong this window by rapidly establishing circulatory and respiratory support through venoarterial extracorporeal membrane oxygenation (VA-ECMO). According to the Extracorporeal Life Support Organization (ELSO), ECPR is defined as the rapid initiation of VA-ECMO in patients with refractory cardiac arrest who do not achieve sustained return of spontaneous circulation (ROSC) through conventional resuscitation.^1^

ECPR has become an established rescue strategy for refractory cardiac arrest in both in-hospital (IHCA) settings and selected out-of-hospital cases (OHCA) referred to specialized centers. Outcomes after cardiac arrest differ substantially between these settings. Patients with IHCA typically experience better survival and neurological outcomes than those with OHCA, primarily due to earlier recognition, immediate high-quality CPR, and rapid access to advanced interventions such as defibrillation and invasive monitoring. Conversely, OHCA is frequently associated with delays in recognition and treatment, prolonged no-flow and low-flow intervals, and variability in prehospital care. Timely intervention and rapid access to advanced therapies remain critical determinants of survival and neurological recovery.

Despite these advantages, IHCA remains a significant clinical challenge. In the United States alone, more than 290,000 adults experience in-hospital cardiac arrest annually,^2^ with persistently high mortality and substantial risk of neurological injury among survivors. ECPR aims to restore systemic perfusion when conventional CPR fails, preserving end-organ function while the underlying cause is identified and treated. Observational studies and registries indicate that, for certain patients such as those with witnessed arrest, reversible causes, and short low-flow times, ECPR may improve survival compared with conventional resuscitation.^3,4^

ECPR use has grown worldwide, but supporting evidence remains limited and varied. Most data come from specialized centers in North America, Europe, and East Asia. Starting the ECPR program requires more than ECMO technology alone. Recommendations highlight the need for strong institutional commitment, multidisciplinary coordination, administrative involvement, feasibility assessment, and clear patient selection criteria.^1^ These programs demand trained staff, dedicated infrastructure, and ongoing experience to ensure rapid response. Successful ECPR programs are usually based on established ECMO centers with experienced teams, protocols, and continuous training. ECPR is the most complex layer of advanced extracorporeal support—a "cherry on top" of a mature ECMO program. Meeting these requirements is challenging for many Latin American healthcare systems, where few centers have the necessary structure, experience, and resources.

In this context, Burgos et al.^5^ deliver valuable insight into the implementation of ECPR in a Latin American setting. In their single-center retrospective cohort, the authors report outcomes of adult patients who underwent VA-ECMO for refractory in-hospital cardiac arrest over nine years at a high-complexity cardiovascular center in Argentina. Although the cohort is relatively small, including fifteen patients, the reported results are notable. The investigators observed successful ECMO weaning in 60% of cases and an in-hospital survival of 46%, with excellent neurological outcomes among survivors and complete conditional survival during follow-up.

These results align with those reported in international registries, where survival after ECPR typically ranges between 30% and 40%.^6^ Notably, these outcomes were achieved in a healthcare environment with fewer resources than those in high-income countries, reinforcing the idea that program success depends not only on technology but also on organization and system efficiency. A key strength of the study is its detailed description of the infrastructure supporting the ECPR program. The authors describe a multidisciplinary ECMO team, available at all hours, that includes cardiovascular surgeons, cardiologists, intensivists, perfusionists, nurses, and other professionals. This organizational structure allowed the implementation of standardized protocols for patient selection, cannulation, anticoagulation management, and post–resuscitation care. System-based organization is increasingly seen as a main factor in extracorporeal resuscitation outcomes. Time to ECMO flow, or low-flow duration, is a strong predictor of survival and neurological recovery.^7^ This interval can be minimized only with well-practiced institutional logistics.

The outcomes reported by Burgos et al.^5^ should be interpreted with caution. The retrospective design, absence of a control group, and small sample size limit definitive conclusions regarding the comparative effectiveness of ECPR. In addition, the cohort was highly selected, including only patients with witnessed arrest, a shockable initial rhythm, and a likely cardiac etiology—factors independently associated with better outcomes regardless of the resuscitation strategy.

The relatively high rates of complications, including bleeding, infection, and acute kidney injury, underscore the complexity of ECPR management. Although these complications are expected and consistent with previous reports, they emphasize the importance of careful patient selection and meticulous multidisciplinary care to ensure that the benefits of ECPR outweigh its risks.

Beyond clinical outcomes, this study offers important insights for the development of ECPR programs in Latin America and other resource-limited regions. Advanced circulatory support is often perceived as inaccessible in low- and middle-income settings due to cost and infrastructure requirements. However, the experience presented by Burgos et al.^5^ suggests that well-structured programs in referral centers can overcome some of these barriers. Concentration of expertise, team training, and implementation of standardized protocols may enable selected centers to deliver advanced therapies with adequate outcomes.

In summary, Burgos et al.^5^ demonstrate the feasibility of ECPR in a Latin American setting. Despite limitations inherent to a small observational study, their findings suggest that outcomes comparable to international standards can be achieved in specialized centers with structured ECMO programs, even in resource-constrained environments. As extracorporeal resuscitation evolves, progress will depend not only on technological advances but also on the development of coordinated systems of care, rigorous patient selection, and sustained institutional commitment.

Ultimately, ECPR should be viewed not only as a technological intervention but also as a system-based therapy that integrates rapid diagnosis, expert resuscitation, and multidisciplinary care.

The experience described in this study emphasizes a fundamental lesson for emerging ECPR programs: although technology and resources are important, the true drivers of success are leadership, organization, training, and institutional commitment. In the end, it is not all about the money—it is about building systems that deliver excellence when every minute matters.
